# In Vitro and In Vivo Antioxidant Activities of Polysaccharides Isolated from Celluclast-Assisted Extract of an Edible Brown Seaweed, *Sargassum fulvellum*

**DOI:** 10.3390/antiox8100493

**Published:** 2019-10-18

**Authors:** Lei Wang, Jae Young Oh, Jin Hwang, Jae Young Ko, You-Jin Jeon, BoMi Ryu

**Affiliations:** 1 Department of Marine Life Sciences, Jeju National University, Jeju Self-Governing Province 63243, Korea; comeonleiwang@163.com (L.W.); ojy0724@naver.com (J.Y.O.); ghkdwls9280@naver.com (J.H.); 2 Marine Science Institute, Jeju National University, Jeju Self-Governing Province 63333, Korea; 3 Material Research Team, Amorepacific R&D Unit, Jeju 63243, Korea; jaeyoung@amorepacific.com

**Keywords:** *Sargassum fulvellum*, polysaccharides, oxidative stress, apoptosis

## Abstract

It has been reported that enzymatic digestion of algae could improve the yield and enhance the biological activity compared to water and organic extraction. Our previous research indicated that Celluclast-assisted extract of *Sargassum fulvellum* (SF) possessed higher carbohydrate content and stronger antioxidant activity compared to water and other enzyme-assisted extracts. In the present study, we evaluated the antioxidant activities of polysaccharides from SF (SFPS) in vitro in Vero cells and in vivo in zebrafish. SFPS was obtained by Celluclast-assisted hydrolysis and ethanol precipitation. Results showed that SFPS contained 74.55 ± 1.26% sulfated polysaccharides and effectively scavenged 1, 1-diphenyl-2-picrylhydrazyl (DPPH), hydroxyl, and alkyl radicals. SFPS significantly and dose-dependently scavenged intracellular reactive oxygen species (ROS) and improved cell viability. Further studies indicated that SFPS reduced apoptotic body formation through downregulation of proapoptotic protein (Bax and cleaved caspase-3) levels and upregulation of antiapoptotic protein (Bcl-xL and PARP) levels in 2,2-azobis(2-amidinopropane) hydrochloride (AAPH)-treated Vero cells. In addition, SFPS showed strong protective effect against AAPH-stimulated oxidative stress in vivo in zebrafish, as demonstrated by the improved survival rate, reduced heart rate, and decrease in ROS, cell death, and lipid peroxidation levels. These results suggest that SFPS possesses strong in vitro and in vivo antioxidant activity and can be a potential ingredient in the pharmaceutical and cosmeceutical industries.

## 1. Introduction

Polysaccharides are the most abundant natural compound produced by algae. Algal polysaccharides are used in food, pharmaceuticals, cosmeceuticals, and other products for human consumption [1]. Various extraction methods have been used to extract soluble components from algae, such as traditional water extraction and organic solvent extraction [2,3,4]. However, these extraction methods are usually associated with higher temperature, longer extraction time, and lower extraction efficiency (the yield varies from 8% to 30%) [5]. The main reason for this is the cell wall polysaccharides in algae, such as cellulose, mannans, xylans, and the matrix phase of water-soluble polysaccharides, which limits the active compound released from the algal cell [6]. In particular, cellulose, the insoluble carbohydrate polymer that is present as hydrogen-bonded crystalline fibers, is the main structural component of the algal cell wall, and it is extremely difficult to degrade [7]. Therefore, an extraction method that can degrade the algal cell wall could effectively increase the yield as well as improve the polysaccharide content in the extract. 

In recent decades, many new extractive techniques have been utilized, such as microwave-assisted extraction, ultrasonic-assisted extraction, and enzyme-assisted extraction. Enzyme-assisted extraction has frequently been applied to extract bioactive compounds from algae because of its high catalytic efficiency and specificity as well as its mild reactive condition [8]. In addition, many studies have suggested that enzymatic digestion of algae not only improves the extraction yield but also enhances the biological activity compared to water and organic extraction [6,9,10,11]. Charoensiddhi et al. reported that enzyme-assisted extraction improved the antioxidant effects of *Ecklonia radiate* extracts [12]. Siriwardhana et al. reported that enzymatic hydrolysis could effectively extract antioxidant compounds from the edible brown seaweed, *Hizikia fusiformis* [13].

Oxidative stress is related to the development of cancer, inflammation, diabetes, obesity, Parkinson’s disease, Alzheimer’s disease, aging, and other diseases [14,15,16,17,18]. It reflects an imbalance between reactive oxygen species (ROS) generation and scavenging. Generally, the amount of ROS generated by normal metabolism can be scavenged by the cellular endogenous antioxidant system [19,20]. However, excessive environmental stresses, such as ultraviolet irradiation, fine dust particles, and chemicals, can cause an abnormal ROS production, which leads to several diseases [21]. Therefore, antioxidant components that possess strong ROS scavenging activity and low or no toxicity may be ideal candidates to develop a therapeutic agent against oxidative stress-related diseases.

Natural compounds possess various bioactivities and have been applied to pharmaceutical and cosmetic areas for a long time [22]. Marine algae-derived compounds, such as polysaccharides, polyphenols, pigments, and sterols, possess various bioactivities, including anti-inflammatory, antitumor, antihypertension, antioxidant, antiobesity, and antidiabetes activities [23,24,25,26]. Marine algae are especially rich in polysaccharides, which generally comprise alginate, carrageenan, and fucoidan [27]. The algal polysaccharides possess potent bioactivities because of their unique physicochemical properties, such as high content of fucose, galactose, uronic acid, and sulfate [28,29]. It has been reported that galactose, fucose, mannose, and sulfate contents are associated with antioxidant activities [30,31]. Thus, algal polysaccharides that are rich in these compositions may possess antioxidant potential.

*Sargassum fulvellum* (*S. fulvellum*) is an edible brown alga, which is consumed as food, herb medicine, and food additive in Asian countries, including China, Korea, and Japan, for a long time [32]. *S. fulvellum* contains various bioactive compounds, especially polysaccharides. Fujihara et al. (1984) isolated polysaccharide from *S. fulvellum* and evaluated its antitumor activity [33]. Chen et al. isolated sulfated polysaccharide fraction from *S. fulvellum* and investigated its immune-stimulating activity [34]. Our previous study investigated the protective effect of enzyme-assisted extracts of *S. fulvellum*, and the results indicated that Celluclast-assisted extract of *S. fulvellum* possessed high carbohydrate content and showed strong antioxidant activity [35]. However, the antioxidant activity of polysaccharides from Celluclast-assisted extract of *S. fulvellum* has not been elucidated. Therefore, in the present study, we investigated the antioxidant activity of polysaccharides from *S. fulvellum* in vitro in Vero cells and in vivo in zebrafish.

## 2. Methods

### 2.1. Alga Material and Extraction 

**Figure 1:**
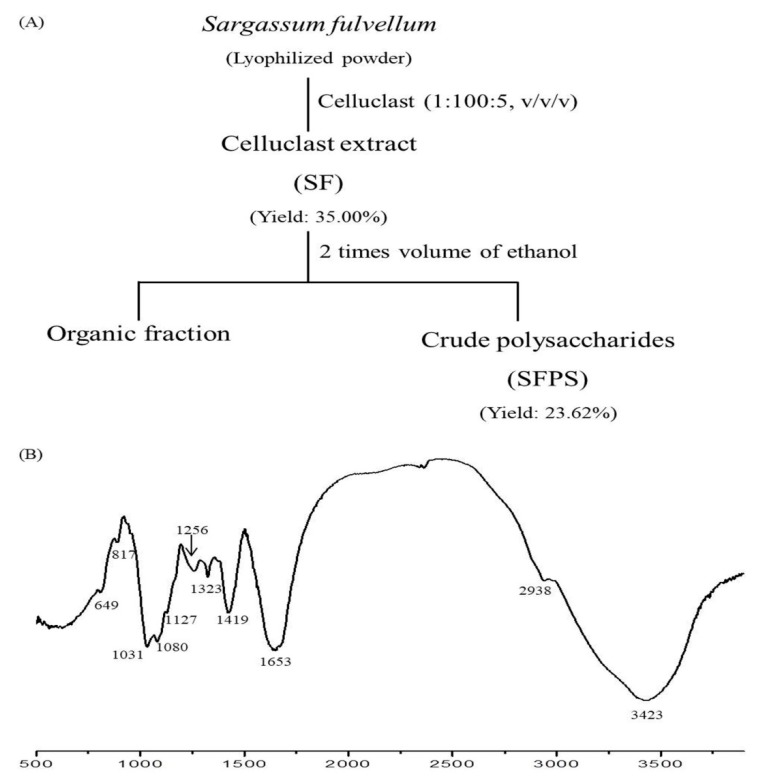
Preparation and characterization of SFPS. (**A**) Extraction protocols; (**B**) FTIR spectrum of SFPS.

*S. fulvellum* was collected in July 2017 from the coastal area of Jeju Island, South Korea. Seaweed was washed by tap water and freeze-dried. The protocol of Celluclast-assisted extraction is described in Figure 1A. In brief, the lyophilized seaweed powder was hydrolyzed by Celluclast (Sigma, St. Louis, MO, USA, ≥700 units/g). A reaction mixture (pH 4.5, 1 L) containing distilled water (999.5 mL), Celluclast (0.5 mL), and lyophilized seaweed powder (10 g) was reacted at 50 °C for 24 h with agitation (120 rpm). After reaction, the enzyme was inactivated by heating at 100 °C for 10 min, and the pH of the reaction mixture was adjusted to 7 by 1 M NaOH. The Celluclast extract of *S. fulvellum* (henceforth referred to as SF) was precipitated by 95% ethanol (2 L). The precipitates that were collected were thought to be the crude polysaccharides of *S. fulvellum* (henceforth referred to as SFPS).

### 2.2. Analysis of Chemical Component 

The total carbohydrate and phenolic contents of SF and SFPS were measured according to the procedures in AOAC Official Methods for Analysis [36]. The sulfate contents of SF and SFPS were determined by the BaCl_2_ gelatin method [37]. The neutral sugar content of the samples was determined by high-performance anion-exchange chromatography with pulsed amperometric detection (HPAE–PAD) following the procedure described in our previous study [4].

### 2.3. Characterization of SFPS by Fourier-Transform Infrared Spectroscopy (FTIR) 

FTIR spectra of the SFPS were analyzed using an FTIR spectrometer (Nicolet 6700; Thermo Scientific, MA, USA). The SFPS powder was homogenized with potassium bromide powder and then pressed into pellets for FTIR measurement in the frequency range of 500–4000 cm^−1^.

### 2.4. In Vitro Analysis

#### 2.4.1. Evaluation of Free Radical Scavenging Abilities of SF and SFPS

The free radical scavenging abilities of SF and SFPS were determined using an ESR spectrometer (JES-FA machine; JOEL, Tokyo, Japan) following the protocols described by Wang et al. [19]. 

#### 2.4.2. Cell Culture

The monkey kidney fibroblasts (Vero cells, KCLB, Seoul, Korea) were subcultured in RPMI-1640 (100 μg/mL of streptomycin, 10% heat-inactivated fetal bovine serum (FBS), and 100 unit/mL of penicillin) and seeded in a 24-well plate (1 × 10^5^ cells/mL) for experiments. 

#### 2.4.3. Determination of the Effects of SFPS in AAPH-Induced Vero Cells

To measure the intracellular ROS levels of AAPH-stimulated Vero cells, cells were seeded and incubated for 24 h. Cells were treated with SFPS (25, 50, and 100 μg/mL) for 1 h. After incubation, AAPH (10 mM) was added to the wells, followed by incubation for 1 h. Finally, the intracellular ROS levels of AAPH-stimulated cells were detected by 2,7-dichlorofluorescein diacetate (DCFH-DA) assay [38].

In order to measure the effects of SFPS against AAPH-induced cellular damage, the viability and the nuclear morphology of AAPH-induced cells were evaluated. Vero cells were treated with SFPS and stimulated with AAPH for 24 h. The cell viability was measured by MTT assay, and the apoptosis level of cells was analyzed by Hoechst 33342 staining assay [16,17,18]. 

Furthermore, expression levels of the key apoptosis-related proteins, including Bax, Bcl-xL, cleaved caspase-3, and PARP, were investigated by Western blot analysis. Vero cells were treated with SFPS and stimulated by AAPH for 24 h. The cells were harvested and lysed. The protein levels of cell lysates were measured by a BCA^TM^ kit (Thermo Scientific, Rockford, MA, USA). Western blot analysis was carried out based on the method described by Wang et al. [24].

### 2.5. In Vivo Analysis

#### 2.5.1. Application of AAPH and/or SFPS to Zebrafish Embryos

Adult zebrafish were maintained according to a previous study [39]. Approximately 7–9 h post-fertilization (hpf), the zebrafish embryos (12-well plate, 15 embryos/well) were incubated in embryo medium containing 25, 50, and 100 μg/mL of SFPS for 1 h. Then, 15 mM AAPH was added to each well, and the embryos were incubated with AAPH until 24 hpf. The zebrafish experiment received approval from the Animal Care and Use Committee of the Jeju National University (Approval No. 2017-0001).

#### 2.5.2. Measurement of Heart Rate, ROS Generation, Cell Death, and Lipid Peroxidation in Zebrafish

The survival rate and the heart rate of zebrafish was measured based on the protocol described by Sanjeewa et al. [4]. The ROS level, cell death, and lipid peroxidation were measured in live zebrafish using DCFH-DA, acridine orange, and diphenyl-1-pyrenylphosphine (DPPP) staining at 3 days post-fertilization (dpf) [39]. 

### 2.6. Statistical Analysis

Data are expressed as the mean ± standard error (SE, *n* = 3). One-way ANOVA test (SPSS 12.0) was used to statistically compare the mean values of each treatment. Significant differences between the means of parameters were determined by Duncan’s multiple range tests, and *p* < 0.05 and *p* < 0.01 were considered significantly different. * *p* < 0.05, ** *p* < 0.01 as compared to the AAPH-stimulated group and ^##^ *p* < 0.01 as compared to the control group.

## 3. Results and Discussion

### 3.1. Chemical Composition and Structural Characterization of SFPS 

Our previous study had hydrolyzed *S. fulvellum* using 10 different enzymes and evaluated the antioxidant activities of the enzymatic extracts. The results suggested that Celluclast-assisted extract possesses higher carbohydrate content and stronger antioxidant activity compared to water and other enzyme-assisted extracts [35]. Therefore, in the present study, Celluclast-assisted extraction was selected to prepare polysaccharides from *S. fulvellum.*

In this study, *S. fulvellum* was hydrolyzed by Celluclast and then precipitated by ethanol to separate the crude polysaccharides. The composition of SF and SFPS was then investigated. The yield of SF was 35.00 ± 0.00%, and the yield of SFPS was 23.60 ± 2.01%. As Table 1 shows, SF contained 1.78 ± 0.05% of phenolic content, 32.50 ± 4.00% of carbohydrate content, and 0.37 ± 0.07% of sulfate content, while SFPS contained 0.77 ± 0.04% of phenolic content, 73.54 ± 1.20% of carbohydrate content, and 1.01 ± 0.06% of sulfate content. Taken together, SF and SFPS contained 32.87 ± 4.07% and 74.55 ± 1.26% sulfated polysaccharides, respectively. These results indicate that phenolic constituents were removed during precipitation, while the carbohydrate and sulfate content was concentrated. SFPS contained much higher sulfated polysaccharides content (73.54 ± 1.20%) than SF. Furthermore, the contents of monosaccharide (fucose, arabinose, glucose, galactose, and xylose) of SF and SFPS were determined. As Table 1 shows, SF contained high glucose (48.02%), but SFPS contained high fucose, galactose, and xylose. Previous research has suggested that fucose, galactose, and xylose are antioxidant activity-related monosaccharaides [22,31]. As SFPS contained high amount of fucose, galactose, and xylose, it may possess strong antioxidant activities. 

The FTIR spectra of SFPS are shown in Figure 1B with its major absorbance peaks. The absorbance peak at 3423 cm^−1^ was the characteristic stretching vibration peak of O–H, and the peak at 2938 cm^−1^ was the characteristic stretching vibration peak of C–H. The characteristic peak at 1653 cm^−1^ was assigned to H–O–H, which demonstrated the presence of moisture in the samples [40]. In addition, absorption peaks at 1256 cm^−1^ and 817 cm^−1^ were assigned to asymmetric stretching vibration of S=O and C–O–S, respectively [4]. These results support that SFPS is sulfated polysaccharides.

It is well known that sulfated polysaccharides from marine algae possess strong free radical scavenging activities [20,22,27]. As Table 2 shows, SF and SFPS showed strongest alkyl radical scavenging activity compared to the other two radicals (DPPH and hydroxyl radicals). Besides, a lower IC_50_ for alkyl radical scavenging activity was observed for SFPS (0.86 ± 0.01 mg/mL) compared to SF (1.03 ± 0.03 mg/mL). AAPH has been used as a free radical generator to produce alkoxyl and alkyl peroxyl radicals for antioxidant studies [41,42]. Therefore, we decided to use AAPH to induce oxidative stress to evaluate the protective effect of SFPS against oxidative stress in vitro in Vero cells and in vivo in zebrafish.

### 3.2. Protective Effect of SFPS on AAPH-Induced Oxidative Stress In Vitro in Vero Cells 

Sulfated polysaccharides from seaweeds possess strong antioxidant activity [43,44,45,46]. As noted above, SFPS contained a high content of sulfated polysaccharides (74.55 ± 1.26%). In addition, there were high amounts of antioxidant-related monosaccharides, such as fucose, galactose, and xylose, in their structures. Moreover, SFPS were found to possess strong free radical scavenging activity, especially for alkyl radical. These results indicated that SFPS had antioxidant activity potential, and it was hence used for further study.

**Figure 2:**
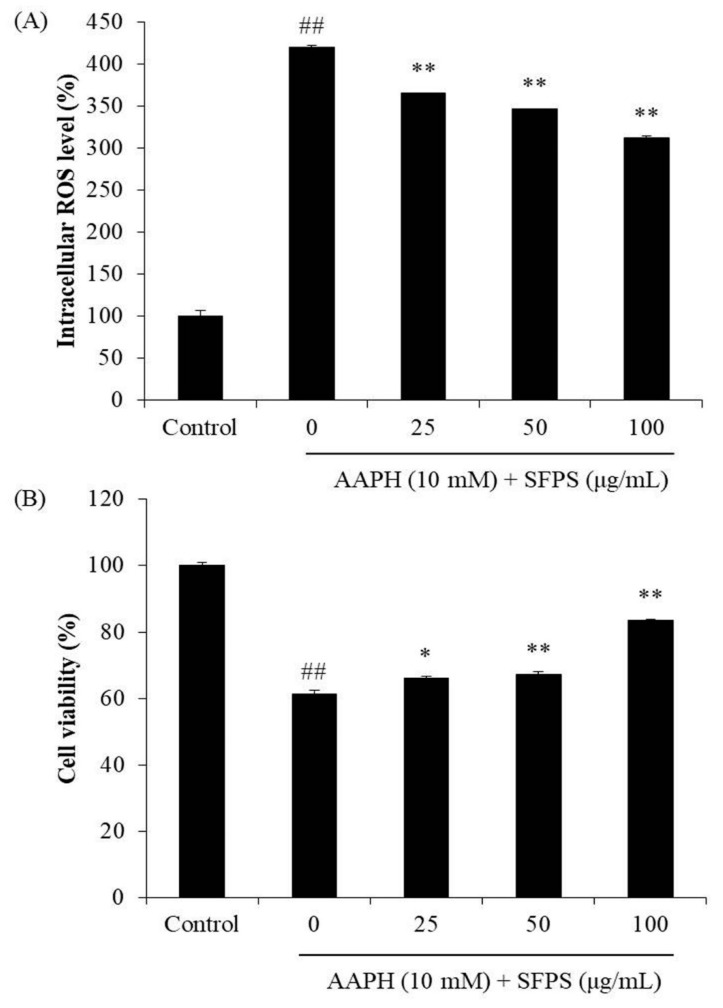
Protective effect of SFPS against AAPH-induced intracellular reactive oxygen species (ROS) production (**A**) and cell death (**B**) in Vero cells. Intracellular ROS level was measured by 2,7-dichlorofluorescein diacetate (DCFH-DA) assay, and cell viability was determined by MTT assay. The data are expressed as means ± standard error (SE) (*n* = 3). *p < 0.05, ** *p* < 0.01 as compared to the AAPH-treated group and ^##^ *p* < 0.01 as compared to the control group.

As shown in Figure 2A, AAPH significantly increased the level of intracellular ROS, whereas SFPS remarkably decreased the intracellular ROS level in a dose-dependent manner. In addition, AAPH decreased cell viability to 61.42%, while SFPS increased cell viability to 66.01%, 67.34%, and 83.62% at the concentration of 25, 50, and 100 μg/mL, respectively (Figure 2B).

**Figure 3:**
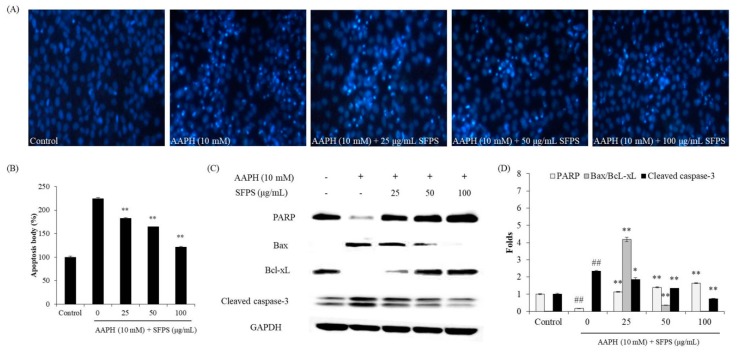
The protective effects of SFPS against AAPH-induced apoptosis in Vero cells. (**A**) Morphology of the Hoechst-stained Vero cells; (**B**) relative apoptotic body level; (**C**) expression levels of apoptosis-related proteins; (**D**) relative amounts of PARP, Bax, Bcl-xL, and cleaved caspase-3. The apoptotic body formation was observed using a fluorescence microscope after Hoechst 33342 staining. The relative amounts of PARP, Bax, Bcl-xL, and cleaved caspase-3 were compared with GAPDH. The relative apoptosis and proteins levels were measured using Image J software. The data are expressed as means ± SE (*n* = 3). ** *p* < 0.01 as compared to the AAPH-treated group and ^##^ *p* < 0.01 as compared to the control group.

Generally, cell death occurs through three major routes: apoptosis, necrosis, and autophagy. Apoptosis is an intrinsic cellular suicidal mechanism regulated by a complex network of signaling pathways, such as Bax, Bcl, PARP, and caspase pathway [47]. As Figure 3A,B shows, AAPH significantly induced apoptosis body formation. However, SFPS remarkably and dose-dependently reduced apoptosis body formation in AAPH-induced Vero cells. Furthermore, AAPH increased the expression of proapoptotic proteins (cleaved caspase-3 and Bax) and decreased the antiapoptotic protein (PARP and Bcl-xL) expression. In contrast, SFPS not only reduced Bax and cleaved caspase-3 levels but also improved PARP and Bcl-xL levels in AAPH-treated Vero cells (Figure 3C,D). Both effects were dose-dependent. These results demonstrate that SFPS possesses in vitro protective effect against AAPH-induced cell damage by regulation of apoptotic-related signaling pathways through ROS clearance. 

### 3.3. Protective Effect of SFPS on AAPH-Induced Oxidative Stress In Vivo in Zebrafish

Zebrafish is a popular animal model in toxicological and pharmacological studies, especially in chemical toxicity and drug discovery. Our previous studies indicated that AAPH significantly induced oxidative stress in zebrafish [31]. In addition, AAPH-stimulated zebrafish model was successfully used to evaluate the antioxidant activity of algal polysaccharides in our previous studies [48]. Kim et al. (2014) investigated the antioxidant effect of fucoidan isolated from *Ecklonia cava* [48]. The results indicated that the fucoidan effectively protected against AAPH-induced oxidative stress in zebrafish, which displayed increased survival rate and improved heart rate as well as reduced ROS, cell death, and lipid peroxidation [48]. Therefore, AAPH-induced zebrafish were used as the model to investigate antioxidant activity of SFPS.

**Figure 4:**
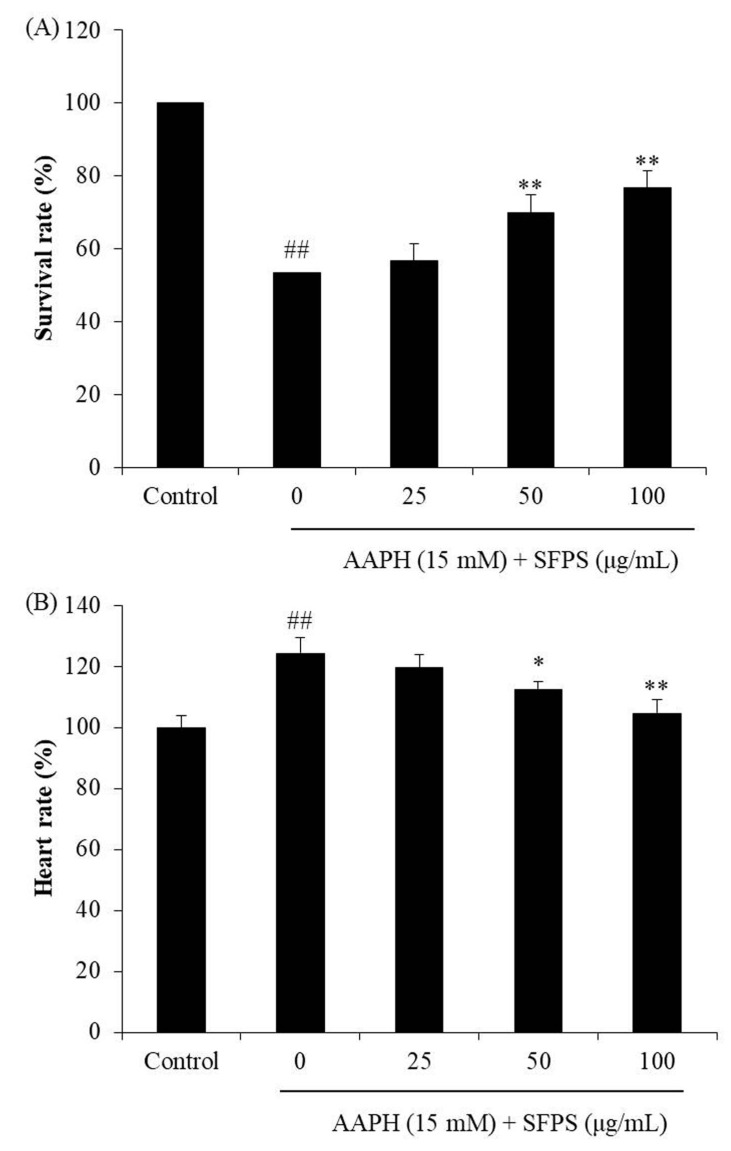
The survival rates and heart rates of zebrafish after pretreatment with SFPS and/or treatment with AAPH: (**A**) survival rate and (**B**) heart rate. The data are expressed as means ± SE (*n* = 3). *p < 0.05, ** *p* < 0.01 as compared to the AAPH-treated group and ^##^ *p* < 0.01 as compared to the control group.

**Figure 5:**
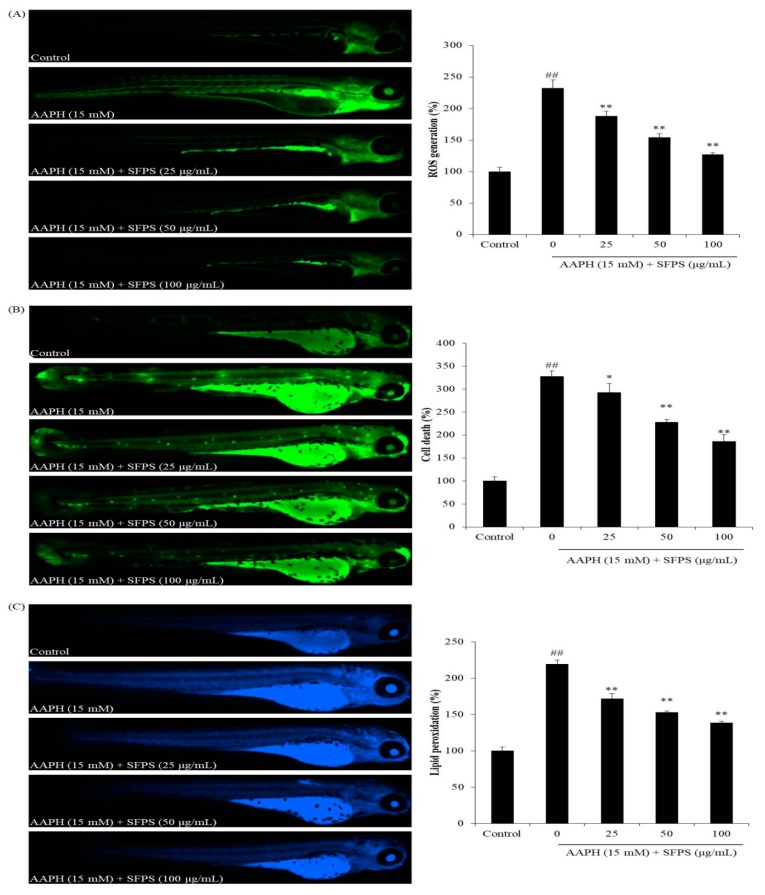
The protective effects of SFPS on AAPH-stimulated oxidative stress in zebrafish. (**A**) ROS generation; (**B**) cell death; and (**C**) lipid peroxidation. ROS, cell death, and lipid peroxidation levels were measured by Image J software. The data are expressed as means ± SE (*n* = 3). *p < 0.05, ** *p* < 0.01 as compared to the AAPH-treated group and ^##^ *p* < 0.01 as compared to the control group.

As Figure 4 shows, the survival rate of AAPH-treated group was 53.33%, and the heart rate was 124.07% compared to the control group (100%). In contrast, the survival rates of SFPS-pretreated groups were significantly increased, and the heart rates were remarkably decreased to the normal level. As Figure 5A shows, intracellular ROS production in AAPH-treated zebrafish significantly increased to 232.27% compared to those not treated with AAPH. However, SFPS treatments at the concentration of 25, 50, and 100 μg/mL decreased intracellular ROS level to 187.73%, 154.26%, and 126.89%, respectively. In addition, AAPH significantly induced cell death, but SFPS significantly decreased AAPH-induced cell death in a dose-dependent manner (Figure 5B). Furthermore, as Figure 5C shows, AAPH significantly increased lipid peroxidation level to 219.28% compared to the control group. However, SFPS decreased lipid peroxidation levels to 171.72%, 152.83%, and 138.45% at the concentration of 25, 50, and 100 μg/mL, respectively. Previous reports have suggested that overproduction of ROS causes the destruction of cells through damage of essential macromolecules such as DNA, protein, and lipid [22,38]. The present results indicate that AAPH significantly induced ROS production, lipid peroxidation, and cell death. It demonstrates that AAPH-induced cell death was caused by accumulation of essential macromolecules damage, such as lipid peroxidation, which is induced by overproduction of ROS. Therefore, the mechanism of SFPS protected zebrafish against AAPH-induced oxidative stress via scavenging ROS. These results indicate that SFPS possesses strong in vivo antioxidant activity, as demonstrated by the decrease in ROS production, cell death, and lipid peroxidation in zebrafish.

## 4. Conclusions

In conclusion, the above results demonstrate that SFPS contained 74.55 ± 1.26% sulfated polysaccharides and possessed strong free radical scavenging activity. In addition, SFPS acted against AAPH-induced Vero cell damage by regulation of apoptotic-related signaling pathways through ROS clearance. Furthermore, SFPS suppressed ROS production and lipid peroxidation as well as cell death in AAPH-treated zebrafish. These results suggest that SFPS possesses strong in vitro and in vivo antioxidant activity and can be a potential ingredient in the pharmaceutical and cosmeceutical industries.

## Figures and Tables

**Table 1:** Components of Celluclast-assisted extract of *Sargassum fulvellum* (SF) and polysaccharides of SF (SFPS) obtained from *S. fulvellum.*

Sample	SF	SFPS
Phenolic content (%)	1.78 ± 0.05	0.77 ± 0.04
Polysaccharide content (%)	32.50 ± 4.00	73.54 ± 1.20
Sulfate content (%)	0.37 ± 0.07	1.01 ± 0.06
Sulfated polysaccharide	32.87 ± 4.07	74.55 ± 1.26
Proportion of monosaccharide (%)		
Fucose	18.76	26.75
Arabinose	3.05	-
Galactose	16.42	33.77
Glucose	48.02	7.71
Xylose	13.75	31.77

**Table 2:** Free radical scavenging activities of SF and SFPS obtained from *S. fulvellum.*

Sample	Free Radical Scavenging Activity (IC_50,_ mg/mL)		
	DPPH	Alkyl	Hydroxyl
SF	9.25 ± 0.39	1.03 ± 0.03	1.22 ± 0.06
SFPS	6.90 ± 0.66	0.86 ± 0.01	1.14 ± 0.12
